# 964. Effects of Conditioned Media of Mesenchymal Stromal Cells in Skin Defects on in Vivo Models

**DOI:** 10.1093/jbcr/irag033.542

**Published:** 2026-04-06

**Authors:** Ahmed Qaraqra

**Affiliations:** Dr. Jesus Yerena General Hospital, Caracas, Distrito Federal

## Abstract

**Introduction:**

Chronic wounds represent a global health problem due to their delayed recovery. Studies have shown that the mechanism of action of mesenchymal stromal cells (MSC) in tissue regeneration is mainly due to the paracrine effect of their secretome.

**Methods:**

It is an experimental, prospective, descriptive and cross-sectional study, experimental animals (mice) were used, platelet-rich plasma (PRP) was prepared, MSC was cultured, the preparation of conditioned media (CM) of 8 and 16 hours, and in vivo surgical procedure was performed in four groups: untreated wounds, wounds treated with PRP clots, wounds treated with CM for 8 hours and 16 hours respectively. Controls were performed on days 3 and 6.

**Results:**

There was no statistically significant difference in wound closure between day 3 and day 6 after wounding in all groups at the macroscopic level. Histological sections of wounds treated with CM-MSC 16 hours show larger areas of re-epithelialization than the other groups. Wounds treated with CM-MSC for 16 hours show greater expression of PDGF and VEGF without a statistically significant difference in polymorphonuclear infiltration between all groups.

**Conclusions:**

CM-MSC-PRP was effective in the treatment of skin wounds and has great potential to be developed as a new acellular therapeutic approach. 16-hour CMs have better therapeutic effects on skin defects than 8-hour CMs, due to the modification of the components of their secretome.

**Applicability of Research to Practice:**

Promising research, further studies are still needed as it is considered experimental at the moment with no experience in our practice in humans.

**Funding for the study:**

N/A.

